# A pilot study of health and wellness coaching for fibromyalgia

**DOI:** 10.1186/s12891-016-1316-0

**Published:** 2016-11-08

**Authors:** Kevin V. Hackshaw, Marcal Plans-Pujolras, Luis E. Rodriguez-Saona, Margaret A. Moore, Erika K. Jackson, Gary A. Sforzo, C. A. Tony Buffington

**Affiliations:** 1 Internal Medicine and Molecular Biochemistry, The Ohio State University, Columbus, USA; 2 Nestlé-Purina, 3916 Pettis Road, Saint Joseph, 64503 MO USA; 3 Food Science and Technology, College of Food Agriculture and Environmental Science, The Ohio State University, 110 Parker Food Science and Technology Building, 2015 Fyffe Road, Columbus, OH 43210 USA; 4 Wellcoaches Corporation, Wellesley, USA; 5 Institute of Coaching, McLean Hospital, a Harvard Medical School affiliate, Belmont, USA; 6 National Consortium for Credentialing Health & Wellness Coaches, San Diego, USA; 7 Exercise & Sport Sciences, 323 Center for Health Sciences, Ithaca College, Ithaca, NY 14850 USA; 8 Veterinary Clinical Sciences, The Ohio State University College of Veterinary Medicine, Columbus, USA; 9 Division of Immunology/Rheumatology, William Davis Medical Research Center, Wexner Medical Center, The Ohio State University, 480 Medical Center Drive, Columbus, OH 43210-1228 USA

**Keywords:** Fibromyalgia, Healthcare costs, Motivational interviewing, Health behavior intervention, Pain management

## Abstract

**Background:**

The purpose of this study was to test the hypothesis that a health and wellness coaching (HWC)-based intervention for fibromyalgia (FM) would result in sustained improvements in health and quality of life, and reductions in health care utilization.

**Methods:**

Nine female subjects meeting American College of Rheumatology criteria for a diagnosis of primary FM were studied. The HWC protocol had two components, which were delivered telephonically over a twelve-month period. First, each patient met individually with a coach during the 12 month study at the patient’s preference of schedule and frequency (Range:22–32 × 45-min sessions). Coaches were health professionals trained in health and wellness coaching tasks, knowledge, and skills. Second, each patient participated in bimonthly (first six months) and monthly (second six months) group classes on self-coaching strategies during the 12 month study. Prior to the intervention, and after 6 months and 12 months of coaching, the Revised Fibromyalgia Impact Questionnaire (FIQR) was used to measure health and quality of life, and the Brief Pain Inventory-Short Form (BPI) was used to measure pain intensity and interference with function. Total and rheumatology-related health encounters were documented using electronic medical records. Data were analyzed using repeated measures ANOVA.

**Results:**

All nine patients finished the HWC protocol. FIQR scores improved by 35 % (*P* = 0.001). BPI scores decreased by 32 % overall (*P* = 0.006), 31 % for severity (*P* = 0.02), and 44 % for interference (*P* = 0.006). Health care utilization declined by 86 % (*P* = 0.006) for total and 78 % (*P* < 0.0001) for rheumatology-related encounters.

**Conclusion:**

The HWC program added to standard FM therapy produced clinically significant improvements in quality of life measures (FIQR), pain (BPI), and marked reductions in health care utilization. Such improvements do not typically occur spontaneously in FM patients, suggesting that HWC deserves further consideration as an intervention for FM.

## Background

Fibromyalgia (FM) is a member of a class of disorders called “medically unexplained symptoms,” “functional somatic syndromes,” or “central sensitivity syndromes,” that present significant diagnostic and therapeutic challenges to medicine [1]. The estimated prevalence of FM in the US in 2005 was about 2 %, affecting an estimated 5 million adults [2, 3]. Fibromyalgia remains undiagnosed in as many as 3 out of 4 people with the condition, and the time from onset of symptoms to diagnosis averages 5 years, resulting in delayed and potentially suboptimal treatment [2]. The economic impact of FM is enormous; current estimates suggest that as many as 25 % of FM patients in the US receive some form of disability or injury compensation [3, 4]. The estimated socio-economic costs for FM are probably in the tens of billions of dollars when one considers work absenteeism, lost productivity, health care utilization (including medication costs), legal fees and litigation, and other costs. Various reports suggest that overall healthcare costs of FM are more than twice the amount for people without FM [5, 6]. As White et al. concluded, “Effective treatment, through improvements in diagnosis, management, and pharmaceutical intervention, could result in reduced direct and indirect costs” [5].

Two factors that determine the subsequent health and quality of life of FM patients are a positive diagnosis and effective treatment [7]. We recently reported that a positive diagnosis can be provided and objective assessment of disease activity determined for FM patients using infrared microspectroscopy and chemometrics [8]. This approach, which represents an advance over traditional methods of clinical diagnosis, currently is undergoing further validation, refinement and approval before being introduced into clinical practice.

Current treatment options include variable combinations of pharmacological and nonpharmacological treatments such as exercise and cognitive behavioral therapy (CBT) [9]. The European League Against Rheumatism (EULAR) has issued strong recommendations for the use of tricyclic antidepressants, serotonin-noradrenaline reuptake inhibitors, serotonin reuptake inhibitors, gamma-amino butyric acid analogues like gabapentin and pregabalin, and only weak recommendations for non-pharmacological therapies such as aerobic exercise, CBT and combination therapy [10]. These recommendations suffer from lack of head to head comparisons of pharmacological versus non-pharmacological treatments [11]. The absence of strong recommendations for non-pharmacologic treatments is perplexing given that many of these modalities have been shown to be effective in this patient population, and have the potential to provide considerable health care cost savings [12, 13]. A recent meta-analysis found only one pharmacologic treatment (amitriptyline) had a significant effect on as many as three of six core FM dimensions (i.e., pain, sleep disturbance, fatigue, affective symptoms, functional deficit and cognitive impairment), whereas non-pharmacologic therapies routinely had multidimensional targets [14]. A recent trial evaluated 12-month treatment patterns and outcomes for 1700 patients starting new medications for FM. Patients reported approximately 20 outpatient visits annually at baseline and 21.2 visits during the year following initiation of FM specific therapy. Number of days absent from work went from 27.7 to 25.0 after new pharmaceutical therapy, but the number of days in bed and days patients received disability income increased from 38.4 to 40.6, and 96.6 to 98.2, respectively [15]. These disappointing outcomes of FM medications illustrate that there is an unmet need to explore the roles for non-pharmacologic FM therapies to reduce health care costs and improve quality of life, as well as to provide more patients access to these therapies [16].

A recent systematic review has documented the effectiveness of health and wellness coaching (HWC) for a variety of chronic medical conditions [17–20]. The HWC approach employs health professionals trained in patient-centered coaching competencies. These include coaching tasks, knowledge, and skills. Coaching competencies are based upon evidence-based theories of behavior change, self-determination, self efficacy, self regulation, positive psychology, and motivational interviewing [21].

The US-based National Consortium for Credentialing Health & Wellness Coaches completed a best-practices job task analysis to enable a national health and wellness coach certification [22]. HWC helps patients or clients identify a personal vision of thriving mentally and physically while assisting in developing autonomous motivation, improving positive emotions, resources, and self-efficacy, and sustaining changes in mindset and behavior that generate improved health and well-being [23]. HWC behavior change techniques have been applied in specific chronic conditions, and meta-analyses have identified significant improvements in pain, fatigue, depression, anxiety, and stress in a variety of clinical populations [18–20, 24]. The cited literature evaluated HWC in patients with cancer pain, post myocardial infarction, and chronic low back pain. Many of the comorbid symptoms these individuals suffer from are similar to the signs of distress in FM, including fatigue, depression, anxiety and stress. Statistically significant improvement was seen in all settings using validated questionnaires of functional improvement including BPI, which we used in our current study. In addition, telephone coaching has been demonstrated to result in significant clinical improvement [24]. Accordingly, HWC appears to be a treatment strategy that might prove effectivel for patients with FM. Hence the purpose of this study was to explore effects of a HWC-based intervention for subjects with FM on outcomes related to health, quality of life and health care costs as documented by subjective global improvement and health care utilization.

## Methods

All study related activity was conducted after obtaining informed consent and in accordance with The Ohio State University Institutional Review Board.

### Subject recruitment

Ten female subjects meeting American College of Rheumatology (ACR) criteria for a diagnosis of primary FM were recruited from The Ohio State University (OSU) Rheumatology clinics located in Columbus OH. Subjects were approached during routine clinic visits with KVH between September 2013 and December 2013 and asked about their willingness to participate in a wellness coaching program. The first ten individuals who accepted this commitment were enrolled in the studies. No financial inducements were provided to patients. One individual elected to withdraw prior to study initiation due to pregnancy-related concerns about the time commitment. None of the individuals recruited were taking any medications other than FDA approved FM medications. There were no changes (additions or change in dosage) to the patients’ medications during the 1 year study period.

### Inclusion/exclusion criteria

All patients met the following criteria: Age 18 – 65 years with history of FM and meeting current ACR criteria. Excluding patients older than 65 helped minimize the chance that pain complaints could be confounded by comorbid osteoarthritis. Onset of FM required that no preceding “physical trauma” or infection were identified as the primary initiating factor in their FM diagnosis. Patients’ entire healthcare was based at OSU Hospitals.

### Medical evaluation

All subjects received an initial medical evaluation by a board-certified rheumatologist specializing in FM (KVH). Evaluation included medical history and prior family history (specifically to identify and exclude individuals whose disease appeared subsequent to some physical, infectious, or emotional insult (e.g., divorce, death in immediate family, etc.). Medical history evaluated the quality and severity of the individual’s pain using visual analog testing and other scales listed below. Physical examination included weight, blood pressure, body mass index, and quantitation of number of tender points (manual and dolorimetry) as described by the ACR [25, 26].

The Revised Fibromyalgia Impact Questionnaire [27] was used to measure physical functioning, work status, depression, anxiety, sleep, pain, stiffness, fatigue, and well-being. The Brief Pain Inventory-Short Form (BPI) [28] was used to measure pain intensity and interference with function. Both assessments were completed prior to commencement of the coaching protocol, and after 6 and 12 months of coaching. We expected improvements in symptom scores at six and twelve months as the benefits of wellness behaviors were experienced based on prior experiences with the HWC approach.

### Coaching protocol

The FM coaching protocol was delivered telephonically as the coaches and clients did not reside in the same area. There were no in-person coaching sessions. The protocol had two components, biweekly private telephone sessions and 18 group telephone sessions over the 12 month period. All study participants [9] participated in the group sessions. The number of private sessions was determined by the the client, not the coach.

Telephonic coaching has been found to be an effective means for behavior change while also providing a convenience for the patient and clinician. Appel et al. demonstrated that weight loss by over 400 obese, at-risk patients was just as effectively achieved using telephonic coaching compared to a condition including in-person sessions [29].

First, each patient met individually with a coach for 45-min private sessions. The timing and number of coaching sessions was based on patient choice and availability, ranging from once to four times per month, for a total range of 22–32 sessions (Table 1).

**Table 1:** Frequency of individual coaching sessions per patient

	Coach 1	Coach 2	Coach 3	Coach 4
Client 1	32 sessions			
Client 2	30 sessions			
Client 3		22 sessions		
Client 4		23 sessions		
Client 5			27 sessions	
Client 6			32 sessions	
Client 7			24 sessions	
Client 8				28 sessions
Client 9				29 sessions

Second, each patient was scheduled for twice monthly (first six months) and then monthly (second six months) group classes on self-coaching strategies. Attendance at the group classes is shown in Table 2.

**Table 2:** Group coaching sessions with number of participants

A	1	2	3	4	5	6	7	8	9	10	11	12	13	14	15	16	17	18
B	8	7	5	6	5	6	7	5	2	4	6	3	4	5	5	4	3	3

A = Group coaching session. B = Number of participants

Individual sessions were delivered by four health and wellness coaches, who completed a professional coach training program consisting of 125 live hours of training in coaching competencies over at least 18 months. The coaches were health professionals with Bachelor’s and Master’s level degrees in areas such as health promotion or nursing, or had advanced exercise physiology certifications. Coaching was delivered as a facilitative (non-directive) patient-centered process following a standard health and wellness coaching definition set forth in a meta-analysis [23] and using well defined coaching techniques and strategies [30]. Patients consistently worked with only one coach for individual private sessions and developed a personal relationship with that coach over the 12-month period. Patients were encouraged to focus the coaching session on topics that would improve their well-being and quality of life; they received support from their coaches for healthy eating, sleep, relaxation, exercise, self-compassion, and mindfulness activities.

Coach 1 had assigned clients with very active lives (Table 1). All clients (whether they attended group sessions or not) were provided with recordings of the sessions. The kick-off meeting was conducted on December 3, 2012 to provide an introduction to Wellcoaches, the project protocol and the underlying theories such as self-compassion and mindfulness. Led by MM and KVH, all participants and coaches were invited to attend. EKJ, four coaches and five participants joined the call, and those who did not attend were provided with a recording.

Recorded 20–30 minute educational webinars on self-coaching topics were provided to patients, encouraging them to engage in “rewiring their brains,” i.e., their thinking and feeling patterns, and personal wellness habits. Twelve self-coaching topics and the sequence with which they were discussed are listed in Table 3. Self-coaching topics were then explored in group coaching sessions, led by one of the four coaches, over the first six months. In the second six-month period, group coaching sessions further reviewed the twelve topics and how the concepts had been, or could continue to be, practically applied in daily living. Patients also were encouraged to write about their personal learning and progress in a journal.

**Table 3:** Self-coaching topics employed in webinars and group coaching sessions

Month	Topic
1	Self-compassion and Self esteem
2	Taming Frenzy and Mindfulness
3	Focus and Positivity
4	Body Intelligence and Motivation
5	Strengths and Curiosity
6	Creativity and Relationships
7	Self-compassion and Self-esteem
8	Frenzy and Mindfulness
9	Focus and Positivity
10	Body Intelligence and Motivation
11	Strengths and Curiosity
12	Creativity and Relationships

### Health care utilization

Patient total and rheumatology health care encounters were documented using the Integrated Health Information Systems at The Ohio State University Hospitals for the periods 12 months prior to the HWC intervention, during the 12 month period of the intervention, and for 12 months after the intervention. Encounters included any interaction that involved provider or patient care associate (nurse, medical assistant, etc.) that resulted in a documented electronic chart entry. Thus, encounters included, but were not limited to, any and all office visits, telephone exchanges or email messages which would be recorded through OSU’s Medical Center Mychart platform, and refill requests. Total health care encounters included all such encounters involving the totality of a patient’s care at OSU Hospitals. Rheumatology Specific Encounters included subspecialty specific calls and telephone messages, refill requests, etc. that were specific only to a patient’s rheumatology related condition.

### Statistical analysis

Group data were evaluated using repeated-measures one-way ANOVA, except for health care encounters, which were not normally distributed. These data were analyzed using the Friedman non-parametric repeated measures one-way ANOVA test. Numbers in Table 5 are scores. The percent changes from baseline were calculated using the formula (baseline score – 6 (or 12) month score)/baseline score. All data were analyzed using commercial statistical software; [31] *P* < 0.05 was considered to be significant.

## Results

Baseline characteristics of the participants is shown in Table 4.

**Table 4:** Baseline characteristics of subjects

Subject	Ethnicity	Education Level	Age	Weight	Height	BMI	BP	PIS	Tender Points	Marital Status	Work Status	INS	Baseline FIQR
1	W	PC	46	133	5’4	22.8	110/80	41	14/18	M	E	I	50.8
2	W	PC	29	137	5’1	25.9	108/63	27	14/18	M	E	I	40.8
3	W	C	53	123	5’5	20.5	110/71	56	18/18	M	NE	I	53.0
4	W	C	57	160	5’5	26.6	161/99	55	14/18	M	NE	I	74.6
5	W	PC	25	105	5’5	17.5	110/70	27	18/18	M	E	I	39.5
6	W	C	49	143	5’2	26.2	102/78	16	18/18	M	NE	I	44.8
7	W	PC	59	155	5’4	26.6	132/82	19	18/18	D	E	I	26.0
8	W	C	57	130	5’5	21.6	124/94	22	18/18	M	NE	I	57.8
9	W	C	27	113	5’2	20.7	102/60	17	16/18	S	E	I	55.7

Race/Ethnicity – W (White). Work Status – E – Employed outside of the home, NE – not employed. M = Married, S = Single, D = Divorced. INS (Insurance) – I = Commercial Insurance, Education Level: C = College, PC = Post College. PIS = Pain Interference Score

**Fig. 1:**
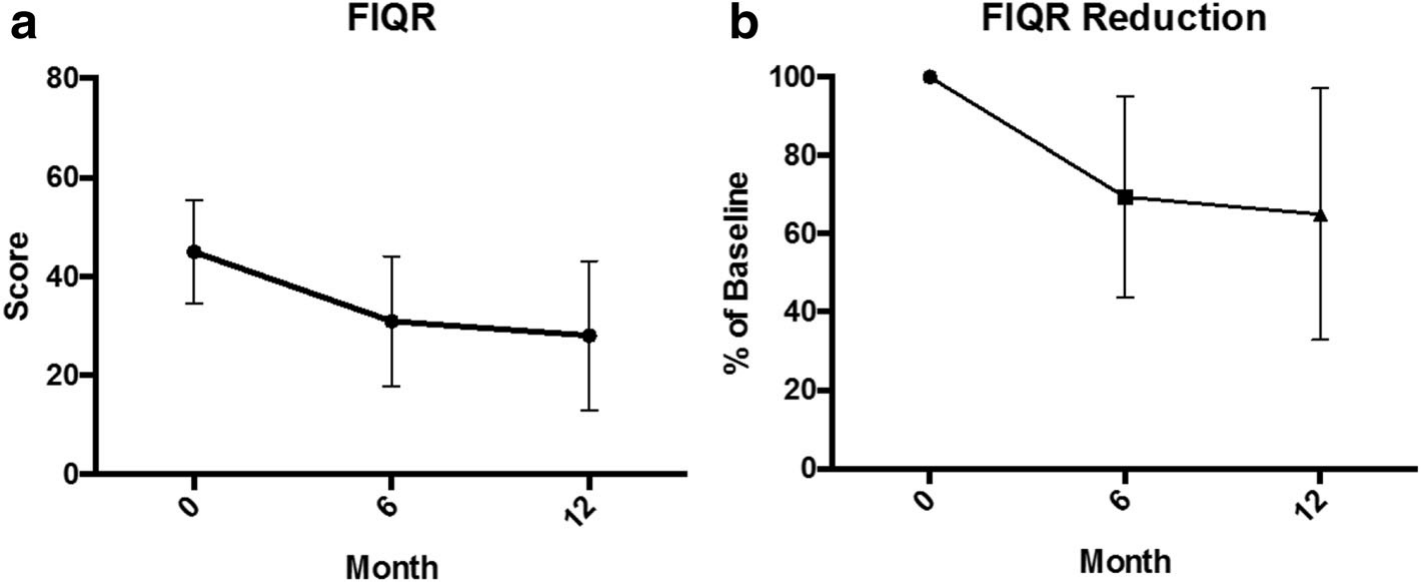
Effect of one year of Health and Wellness Coaching on Revised Fibromyalgia Impact Questionnaire (FIQR) scores. Change in patient scores is presented in Panel A, and Mean ± S.D. reduction in scores is presented in Panel B. Significant improvement in scores (FIQR *P* = 0.001) and reduction of fibromyalgia impact (FIQR reduction *P* = 0.006 were identified) using 1-way repeated measures ANOVA (*n* = 9)

Although all nine patients finished the HWC protocol, complete data sets were available from eight patients because one patient did not return 12-month questionnaires; 12-month values for this patient were imputed to be the mean of the other patients’ 12-month results. Results of HWC on the FIQR are presented in Fig. 1. One-way repeated measures ANOVA revealed a significant reduction in both domains between baseline and the 6 month evaluation, which was sustained at the 12 month evaluation.

Numerical results for the FIQR, BPI, including the severity and interference subscales, are presented in Table 5, and for total and rheumatology-related health care encounters in Table 6. Statistically and clinically significant effects of HWC coaching were identified on all measured parameters. There was no change in number of tender points from baseline to end of study.

**Table 5:** Effects of health and wellness coaching on patient factors [% reduction from baseline]

Measure	0	6	12	P
Revised Fibromyalgia Impact Questionnaire	49.4 ± 13.8^a^	31.0 ± 13.2 [37.2 %]	32.2 ± 12.7 [34.8 %]	0.001
Brief Pain Inventory (BPI)	56.7 ± 18.1	37.0 ± 17.7 [34.7 %]	38.7 ± 20.9 [31.7 %]	0.006
BPI severity	4.8 ± 1.2	3.2 ± 1.6 [33.3 %]	3.3 ± 2.1 [31.3 %]	0.02
BPI interference	4.5 ± 2.3	2.8 ± 2.1 [37.8 %]	2.5 ± 2.2 [44.4 %]	0.002

^a^Mean ± Std. dev

**Table 6:** Effects of health and wellness coaching on patient health care utilization factors [% reduction from baseline]

Measure	12 months prior to intervention	During 12-month intervention	12 months after intervention	P
Health care encounters - total	11 (3, 16)^a^	3 (2, 9) [72.7 %]	1.5 (1, 5) [86.4 %]	0.006^b^
Health care encounters - rheumatology	4.5 (3,6)	2 (2, 3) [55.6 %]	1 (0.25, 1) [77.8 %]	<0.0001^c^

^a^Median (25^th^, 75^th^ percentiles)

^b^Friedman test

Both 6 and 12 month results were significantly different from 0 and not different from each other.

## Discussion

This was a one year pilot study of the effectiveness of a HWC intervention for 9 female clients with FM recruited from a university rheumatology practice. The study resulted in three significant findings. First, we found that complementing usual care with HWC led to a significant decrease in the overall FIQR score, suggesting overall improvement in quality of life. Bennett and colleagues previously suggested that FIQ changes of 14 % or greater from baseline value may be clinically meaningful [32]. We noted improvements of 37 and 35 % at 6 and 12 months, respectively, supporting the beneficial impact of the HWC intervention. Second, we found significant improvements in the BPI, and its severity and interference subscales, at 6 months, which were sustained at 12 months. Third, we identified large, highly statistically significant reductions in both total and rheumatology-related health care encounters recorded in the integrated health information system network.

As stated in the title, this was a pilot study to determine if HWC had any effect on the measured parameters in patients with FM. To our knowledge, this is the first time HWC has been applied to FM, so no sample size calculation could be performed. The present data will be useful for such calculations for future randomized controlled trials comparing HWC to other approaches for treatment for FM. This was the approach recommended by our statistical consultant.

Integrating diverse domains of psychological research and theory, health and wellness coaches help clients consider what it means personally to thrive mentally and physically, and envision a desired future that is fuel for their autonomous motivation [33]. HWC helps clients develop higher levels of positive emotions, which have been shown to improve cognitive function, resilience and immune system function, and to reduce incidence of physical symptoms and disease [34]. The coaching relationship is then designed to leverage enhanced self-motivation and positive emotions to improve self-efficacy. Clients experiment with behavior changes using a growth mindset (focus on learning versus success or failure) tailored to their personal circumstances so that they discover a combination of lifestyle behaviors that they can sustain and that lead to improved health and well-being. The HWC approach can yield significant long-term improvements in health and quality of life, as documented in a recent systematic review [17]. To date, positive HWC outcomes include reduced hemoglobin A1C, weight loss, increased smoking cessation rates, enhanced mood and exercise participation, reduced anxiety post-myocardial infarction, and improved physical and behavioral health, and healthy behavior [17, 35]. Moreover, wellness coaching using private telephone sessions has led to improved health status and health behaviors in patients with chronic conditions [36]. According to the present results, FM also may be effectively treated using HWC.

The HWC approach compares favorably with current pharmacological treatments for FM. For example, Arnold, et al., [37] reported results of a 12 week study comparing duloxetine with placebo in 207 patients (89 % female) with FM. As in the present study, they used the FIQ and the BPI as outcome measures. The results of HWC coaching were comparable to duloxetine for both the FIQR and for severity and interference scales of the BPI. More recently, Gilron, et al., [38] evaluated the efficacy of four different therapeutic regimens for FM; placebo, pregabalin, duloxetine, and pregabalin + duloxetine over a 6 week trial period. The baseline FIQ score was 45.2 ± 1.9. At 6 weeks, the FIQ scores (% improvement) were 42.9 ± 2.3 (5.1 %; placebo), 37.4 ± 2.3 (17.2 %; pregabalin), 36.0 ± 2.4 (20.4 %; duloxetine) and 29.8 ± 2.5 (34.1 %; pregabalin + duloxetine).

Arnold et al., examined FM responder definitions in an attempt to identify those that were most sensitive in identifying response to treatment. After reviewing a number of indices, they concluded that greater than or equal to 30 % reduction in pain and greater than or equal to 10 percent improvement in physical function was most consistent with clinically significant improvements [39]. Both of these threshold were exceeded in our study.

There have been several evaluations of behavioral medicine approaches to FM, including CBT [12, 13, 40, 41] and acceptance and commitment therapy (ACT). Previous studies have reported improvements following interventions that incorporated CBT techniques for patients with FM and related functional somatic syndromes. For example, Fjorback and colleagues reported a significant decrease in total health care utilization with the use of CBT-like techniques and thus the potential to significantly reduce overall health care costs in individuals with these disorders [42]. Luciano and colleagues [43] compared the effectiveness of ACT on pain acceptance as a mediator of treatment outcomes and functional status in FM patients. Patients were randomly assigned to a group–based form of ACT, recommended pharmacological treatment (pregablin + duloxetine) or wait list. The primary end point was functional status measured with the FIQ. Baseline FIQ in the 3 groups were 68.2, 69.0 and 65.9 respectively. After treatment, FIQ scores were 48.7, 63.4 and 67.7 representing 28.6, 8.1 and ~ −2.7 % improvement. At 6 – month follow-up, values were 49.5, 65.1, and 67.4, representing improvements of 27.4 % (ACT), 5.9 % (pregabalin + duloxetine) and −2.3 % (wait list). In contrast, there have been no prior reports to our knowledge of a HWC intervention similar to our design as an approach to treatment of FM. Our results of >35 % FIQR improvement equal or exceed outcomes of current pharmacological treatments and ACT, and support further study of HWC as an effective complementary intervention for patients with FM.

The high economic burden of FM in western countries has been well documented. Multiple studies have shown escalating annual costs in FM patients as opposed to other control groups of patients. In one study, health care utilization, medication and work loss estimates for FM were $5163, relative to $2486 in overall employee samples from 1998 data [44]. In another study, Berger, et al., [45] reported that FM patients were more likely to have received various combinations of pain-related medication. They also found that the mean number of physician appointments was 4 times higher, and mean total direct costs 3 times higher, among patients with FM than in a comparison group ($9573 versus $3291). Interestingly, Boonen and colleagues [46] found that FM patients referred for subspecialist care had an even higher utilization of health care resources with more productivity loss and higher average annual total costs than did patients with conditions such as low back pain or ankylosing spondylitis (£7813 versus £3205).

We found a highly statistically significant decreases in health care utilization by the entire group during and following the HWC intervention. We currently are more than 12 months post coaching intervention, and can report that these patient’s provider contact remains minimal. These observations suggest sustained improvement for an additional 12 months, perhaps as a function of the individuals internalizing self – coaching skills that have been sustained following the year of HWC intervention. Thus, our findings, albeit in a very small cohort, strongly suggest the opportunity for HWC to significantly increase health care cost savings over time, and provide a strong impetus for further studies of HWC in a larger cohort using more stringent tools to analyze the cost effectiveness of the intervention from both health care and overall societal impact perspectives. For example, our institution utilizes multiple patient call centers staffed by nurses in a ratio of 1 nurse per 3 physicians to receive and log in phone calls from patients during the workday. The rheumatology call center averages 210 calls per workday. Although the cost of telephone encounters has not yet been quantified, each phone call at our institution during the most recent three month monitoring period averaged 3 minutes and 18 s. Although length of phone calls by specific disease state are not available, there is unanimous agreement among our call center staff that individuals with FM who call in with health related concerns require a much lengthier discussion than do individuals who might call in with osteoarthritis or low back pain-related questions, for example.

In our pilot study, conclusions were based on encounters tabulated through our integrated health information service for those subjects who received the entirety of their health care in OSU Hospitals System. We did not address economic impact outside health care, such as productivity or absenteeism. Further studies with a larger cohort will be required to assess the non health care impacts of HWC further, [42] and to conduct a detailed cost analysis of the intervention to determine the overall economic impact, following the approach to costing procedure described by Luciano and colleagues [43].

The clients in our study averaged 24 individual coaching sessions at a cost ($75 session fee paid to coaches), or approximately $1800 per patient. The group sessions cost approximately $300 per client ($150 fee paid to coach for 18 group coaching sessions), for a total cost of $2100 per patient over the course of one year.

The cost of seeing a health professional 4 times a year would average between $73 dollars and $108 dollars based on whether the Evaluation and Management coding was designated as Level 3 or 4 (https://www.cms.gov/Outreach-and-Education/Medicare-Learning-Network-MLN/MLNProducts/MLN-Publications-Items/CMS1243514.html). If we estimate the average cost at approximately $90 per visit, over the course of one year, total cost of visits would be $360 ($720 for 2 years). This figure does not include cost of medications. In that we noted a decrease in encounters over time by participants in the study makes us optimistic that the HWC intervention could translate into a decrease in overall provider visits, decreased absenteeism at work, greater work productivity and decreased medication utilization. It is intriguing to speculate that positive gains achieved by HWC could potentially be sustained for even longer periods by periodic interventions for the purposes of reinforcement.

The patients were not incentivized financially to participate. They also did not pay for the individual or group sessions. The coaching costs were paid for entirely by the research organizations. In our small cohort, we had excellent compliance with patients attending many sessions over the course of 12 months. We surmise that engagement level was high because patients were deriving personal benefits. We recognize that we had a highly educated cohort in this study. In addition, all of our patients were insured. Future studies will seek to address what sort of adherence rate(s) might be achieved with lower income individuals, or from individuals that may not have the same level of education background as our current cohort.

There are a number of limitations to our pilot study. First of all, there was no way to determine how much “buy in” each subject had for HWC. All subjects participated in one-on-one coaching sessions, ranging from a total of 22 to 32 sessions, but in group sessions, as would be expected in a group of diverse personalities and life circumstances, some patients attended more group sessions than did others, and some were more “vocal” and seemingly more engaged than others. The only way to differentiate the impact of group sessions versus individual sessions would be to design a similar prospective study contrasting 2 group sessions per month versus one-on-one sessions and reevaluate all measurable indicators of impact on FM. Additionally, we were limited in the number of variables that could be tested by the small sample size. For example, the pharmacological aspects of treatment were not tightly controlled in this study. Subjects were basically maintained on “usual care” without major changes to their typical analgesic or neuropathic regimen. This standardized maintenance of usual care was achieved since all individuals were under the management of one of us (KVH). However, with a larger sample size and increased power it would be possible to control and test the effects of medication on efficacy of coaching. In addition, physical activity levels of subjects were not rigorously assessed during this study. It is possible that subjective overall improvement may have been enhanced by increased physical activity on the part of the participants. Future research can evaluate whether subjects make any changes to their overall physical activity level during the course of coaching, perhaps as a result of variable combinations of improved ability to cope with pain levels and a positive overall improvement in pain. It would also be of interest to see if sleep was positively or negatively affected during the course of coaching. Having a set monitoring period with an actimeter for subjects could help to answer these questions.

We cannot determine what effect receiving attention from coaches on a regular basis conveyed on the treatment cohort. Future studies that include two groups of demographically matched subjects with one group bereft of group coaching on self coaching techniques will help determine whether individual coaching coupled with group coaching is more effective than coaching without it in terms of pain reduction, cost reduction, quality of life indicators, etc. It is our intent to conduct a larger controlled trial that will also compare pharmacological and HWC by themselves and the combined effects of both with the end goal being to develop the most effective, evidence based treatment for individuals with FM by testing combinations of medication and HWC protocols.

## Conclusions

In summary, addition of a HWC program to pharmacologic management of patients with FM therapy produced clinically significant improvements in patient quality of life measures (FIQR), reduction in pain (BPI) severity and interference, and marked reductions in health care utilization. Further studies of these interventions are essential to improve the quality of life of FM patients and to reduce the economic burden of FM on our societies. More emphasis on non-pharmacologic intervention may need to be done in the future to include HWC techniques as part of standard treatment for FM rather than considering them as adjunctive therapy.

## Abbreviations

ACR: American college of rheumatology
ACT: Acceptance and commitment therapy
ANOVA: Analysis of variance
BPI: Brief pain inventory
CBT: Cognitive behavioral therapy
EULAR: European league against rheumatism
FDA: Food and drug administration
FIQR: Revised fibromyalgia impact questionnaire
FM: Fibromyalgia
HWC: Health and wellness coaching
OSU: The Ohio State University

## References

[1] Yunus MB (2015). Editorial review: an update on central sensitivity syndromes and the issues of Nosology and psychobiology. Curr Rheumatol Rev.

[2] Arnold LM, Clauw DJ, McCarberg BH, FibroCollaborative (2011). Improving the recognition and diagnosis of fibromyalgia. Mayo Clin Proc.

[3] Smith HS, Harris R, Clauw D (2011). Fibromyalgia: an afferent processing disorder leading to a complex pain generalized syndrome. Pain Physician.

[4] Wolfe F (1996). The fibromyalgia syndrome: a consensus report on fibromyalgia and disability. J Rheumatol.

[5] White LA, Birnbaum HG, Kaltenboeck A, Tang J, Mallett D, Robinson RL (2008). Employees with fibromyalgia: medical comorbidity, healthcare costs, and work loss. J Occup Environ Med.

[6] Thompson JM, Luedtke CA, Oh TH, Shah ND, Long KH, King S (2011). Direct medical costs in patients with fibromyalgia: cost of illness and impact of a brief multidisciplinary treatment program. Am J Phys Med Rehab.

[7] Clauw DJ (2014). Fibromyalgia: a clinical review. JAMA.

[8] Hackshaw KV, Rodriguez-Saona L, Plans M, Bell LN, Buffington CAT (2013). A bloodspot-based diagnostic test for fibromyalgia syndrome and related disorders. Analyst.

[9] Arnold LM, Gebke KB, Choy EH (2016). Fibromyalgia: management strategies for primary care providers. Int J Clin Pract.

[10] Carville SF, Arendt-Nielsen L, Bliddal H, Blotman F, Branco JC, Buskila D (2008). EULAR evidence-based recommendations for the management of fibromyalgia syndrome. Ann Rheum Dis.

[11] Nuesch E, Hauser W, Bernardy K, Barth J, Juni P (2013). Comparative efficacy of pharmacological and non-pharmacological interventions in fibromyalgia syndrome: network meta-analysis. Ann Rheum Dis.

[12] Richmond J, Berman BM, Docherty JP, Goldstein LB, Kaplan G, Keil JE (1996). Integration of behavioral and relaxation approaches into the treatment of chronic pain and insomnia. JAMA.

[13] Williams D, Cary M, Glazer L, Rodriguez A, Clauw D (2000). Randomized controlled trial of CBT to improve functional status in fibromyalgia. Am Coll Rheum.

[14] Perrot S, Russell IJ (2014). More ubiquitous effects from non-pharmacologic than from pharmacologic treatments for fibromyalgia syndrome: a meta-analysis examining six core symptoms. Eur J Pain.

[15] Robinson RL, Kroenke K, Williams DA, Mease P, Chen Y, Faries D (2013). Longitudinal observation of treatment patterns and outcomes for patients with fibromyalgia: 12-month findings from the reflections study. Pain Med.

[16] Clauw DJ, Crofford LJ (2003). Chronic widespread pain and fibromyalgia: what we know, and what we need to know. Best Pract Res Clin Rheumatol.

[17] Kivela K, Elo S, Kyngas H, Kaariainen M (2014). The effects of health coaching on adult patients with chronic diseases: a systematic review. Patient Educ Couns.

[18] Thomas ML, Elliott JE, Rao SM, Fahey KF, Paul SM, Miaskowski C (2012). A randomized, clinical trial of education or motivational-interviewing-based coaching compared to usual care to improve cancer pain management. Oncol Nurs Forum.

[19] O’Neil A, Hawkes AL, Atherton JJ, Patrao TA, Sanderson K, Wolfe R (2014). Telephone-delivered health coaching improves anxiety outcomes after myocardial infarction: the ‘ProActive Heart’trial. Eur J Prev Cardiol.

[20] Duijts SF, Faber MM, Oldenburg HS, van Beurden M, Aaronson NK (2011). Effectiveness of behavioral techniques and physical exercise on psychosocial functioning and health‐related quality of life in breast cancer patients and survivors—a meta-analysis. Psycho-Oncol.

[21] Moore M, Phillips E, Hanc J. Organize your emotions, optimize your life: decode your emotional DNA- and thrive. Harvard University Press; 2016.

[22] Jordan M, Wolever RQ, Lawson K, Moore M (2015). National training and education standards for health and wellness coaching: the path to national certification. Global Adv Health Med.

[23] Wolever RQ, Simmons LA, Sforzo GA, Dill D, Kaye M, Bechard EM (2013). A systematic review of the literature on health and wellness coaching: defining a key behavioral intervention in healthcare. Glob Adv Health Med.

[24] Iles R, Taylor NF, Davidson M, O’Halloran P (2011). Telephone coaching can increase activity levels for people with non-chronic low back pain: a randomised trial. J Physiother.

[25] Wolfe F, Clauw DJ, Fitzcharles MA, Goldenberg DL, Katz RS, Mease P (2010). The American College of Rheumatology preliminary diagnostic criteria for fibromyalgia and measurement of symptom severity. Arthritis Care Res.

[26] Wolfe F, Smyth HA, Yunus MB, Bennett RM, Bombardier C, Goldenberg DL, Tugwell P, Campbell SM, Abeles M, Clark P, Fam AG, Farber SJ, Fiechtner JJ, Franklin CM, Gatter RA, Harnaty D, Lessard J, Lichtbroun AS, Masi AT, Mccain GA, Reynolds WJ, Romano TJ, Russell IJ, Sheon RP (1990). The American College of Rheumatology 1990 criteria for the classification of fibromyalgia. Arthritis Rheum.

[27] Bennett RM, Friend R, Jones KD, Ward R, Han BK, Ross RL (2009). The revised fibromyalgia impact questionnaire (FIQR): validation and psychometric properties. Arthritis Care Res.

[28] Cleeland CS (2009). The brief pain inventory user guide.

[29] Appel LJ, Clark JM, Yeh HC, Wang N-Y, Coughlin JW, Daumit G, Miller ER, Dalcin A, Jerome GJ, Geller S, Noronha G, Pozefsky T, Charleston J, Reynolds JB, Durkin N, Rubin RR, Louis TA, Brancati FL (2011). Comparative effectiveness of weight-loss interventions in clinical practice. NEJM.

[30] Moore M, Tschannen-Moran R, Jackson E (2015). Coaching pyschology manual.

[31] GraphPad. Prism version 6.00 for Mac. La Jolla California USA: GraphPad Software.

[32] Bennett RM, Bushmakin AG, Cappelleri JC, Zlateva G, Sadosky AB (2009). Minimal clinically important difference in the fibromyalgia impact questionnaire. J Rheumatol.

[33] Moore M, Tschannen-Moran B. Coaching psychology manual. Wolters Kluwer Health/Lippincott, Williams & Wilkins; 2010.

[34] Moore M, Hammerness P. Organize your mind, organize your life: train your brain to get more done in less time. Harlequin; 2012.

[35] Prochaska JO, Evers KE, Castle PH, Johnson JL, Prochaska JM, Rula EY (2012). Enhancing multiple domains of well-being by decreasing multiple health risk behaviors: a randomized clinical trial. Popul Health Manag.

[36] Dennis SM, Harris M, Lloyd J, Davies GP, Faruqi N, Zwar N (2013). Do people with existing chronic conditions benefit from telephone coaching? A rapid review. Aust Health Rev.

[37] Arnold LM, Lu Y, Crofford LJ, Wohlreich M, Detke MJ, Iyengar S (2004). A double-blind, multicenter trial comparing duloxetine with placebo in the treatment of fibromyalgia patients with or without major depressive disorder. Arthritis Rheum.

[38] Gilron I, Chaparro LE, Tu D, Holden RR, Milev R, Towheed T (2016). Combination of pregabalin with duloxetine for fibromyalgia: a randomized controlled trial. Pain.

[39] Arnold LM, Williams DA, Hudson JI (2012). Development of responder definitions for fibromyalgia clinical trials. Arthritis Rheum.

[40] Keefe FJ (1996). Cognitive behavioral therapy for managing pain. Clin Psychol.

[41] Keefe FJ, Surwit RS, Pilon RN (1980). Biofeedback, autogenic training, and progressive relaxation in the treatment of Raynaud's disease: a comparative study. J Appl Behav Anal.

[42] Fjorback LO, Arendt M, Ornbol E, Walach H, Rehfeld E, Schroder A (2013). Mindfulness therapy for somatization disorder and functional somatic syndromes: randomized trial with one-year follow-up. J Psychosom Res.

[43] Luciano JV, Guallar JA, Aguado J, Lopez-Del-Hoyo Y, Olivan B, Magallon R (2014). Effectiveness of group acceptance and commitment therapy for fibromyalgia: a 6-month randomized controlled trial (EFFIGACT study). Pain.

[44] Robinson RL, Birnbaum HG, Morley MA, Sisitsky T, Greenberg PE, Wolfe F (2004). Depression and fibromyalgia: treatment and cost when diagnosed separately or concurrently. J Rheumatol.

[45] Berger A, Dukes E, Martin S, Edelsberg J, Oster G (2007). Characteristics and healthcare costs of patients with fibromyalgia syndrome. Int J Clin Pract.

[46] Boonen A, van den Heuvel R, van Tubergen A, Goossens M, Severens JL, van der Heijde D (2005). Large differences in cost of illness and wellbeing between patients with fibromyalgia, chronic low back pain, or ankylosing spondylitis. Ann Rheum Dis.

